# Effect of advanced glycation end products on the expression of hypoxia-inducible factor-1α and vascular endothelial growth factor proteins in RF/6A cells

**DOI:** 10.3892/etm.2013.1015

**Published:** 2013-03-15

**Authors:** HUIMING ZHANG, LUOSHENG TANG, SIYING CHEN, YEZHEN YANG, MINGJIAZI CHEN, JING LUO

**Affiliations:** Department of Ophthalmology, The Second Xiangya Hospital, Xiangya School of Medicine, Central South University, Changsha, Hunan 410011, P.R. China

**Keywords:** hypoxia-inducible factor-1α, vascular endothelial growth factor, advanced glycation end products, RF/6A cells

## Abstract

The aim of this study was to investigate the effect of advanced glycation end products (AGEs) on the expression of hypoxia-inducible factor-1α (HIF-1α) and vascular endothelial growth factor (VEGF) proteins in RF/6A cells cultured *in vitro*, and to investigate the association between the expression of HIF-1α and VEGF proteins. RF/6A cells were cultured *in vitro* and treated with AGEs and non-glycated albumin control at various concentrations (0, 50, 100, 200, 400 and 800 mg/l) for 24 h. The expression of the VEGF protein was detected by ELISA, and western blot analysis was used to determine the levels of HIF-1α protein. The expression of HIF-1α and VEGF proteins was significantly higher in the AGE group compared with the non-glycated control group (all P<0.05). With the increase in concentration of AGEs, the expression levels of HIF-1α and VEGF protein increased and reached a maximum at 200 mg/l AGE, then decreased at 400 and 800 mg/l. However this effect was not observed in the non-glycated control groups. There was a positive correlation between the expression of HIF-1α and VEGF (P<0.05). AGEs induced the expression of HIF-1α and VEGF proteins in RF/6A cells in a concentration-dependent manner. AGEs may upregulate the expression of VEGF protein by increasing the levels of HIF-1α protein, demonstrating the potential role of HIF-1α-targeted therapy in neovascularization.

## Introduction

Diabetic retinopathy (DR) is a common blindness-causing retinal vascular disease. The pathogenesis of DR is complex and has yet not been clarified. Advanced glycation end products (AGEs) are a class of complex products. AGEs are the results of a reaction between carbohydrates and free amino groups on proteins. AGEs are toxic and may induce bacteria to undergo mutagenesis. AGEs are formed in excess during aging, diabetes mellitus and renal failure (1). A key characteristic of certain reactive or precursor AGEs is their ability to form covalent crosslinks between proteins which alters their structure and function, as observed in the cellular matrix, basement membranes and vessel-wall components (2). In addition, AGEs are associated with the development of DR. However, the pathogenic mechanisms are poorly defined. Vascular endothelial growth factor (VEGF) is widely recognized as the most influential factor that induces mitosis and regulates the permeability of endothelial cells, and increases vascular permeability. VEGF levels are increased in ischemic and nonischemic diabetic retina, and VEGF is required for the development of retinal and iris neovascularization. In addition, VEGF alone may induce the majority of the concomitant pathology of DR. Intraocular VEGF levels are increased in diabetic patients (3). Furthermore, the specific intravitreous injected inhibition of VEGF may reduce the symptoms of DR (4). Hypoxia-inducible factor-1 (HIF-1) is a key transcription factor, involved in the regulation of intracellular metabolism, which induces the expression of the downstream gene VEGF (5). In addition, HIF-1α plays a pathogenetic role in DR (6). However, the mechanism by which AGEs modulate the expression VEGF and HIF-1α, and the correlation between VEGF and HIF-1α is not clear. With reference to these data, we examined the role of AGEs in the induction of VEGF and HIF-1α expression *in vitro*.

## Materials and methods

### 

#### Materials

Fetal bovine serum (FBS) was purchased from PAA Laboratories (Colbe, Germany). DMSO was obtained from Sigma (St. Louis, MO, USA), anti-mouse polyclonal antibody HIF-1α was purchased from Novus Biologicals (NB100-105; Littleton, CO, USA) and 4% paraformaldehyde was purchased from Wuhan Boster Biological Technology, Ltd. (Wuhan, China). The bicinchoninic acid (BCA) and VEGF ELISA kit were obtained from Beijing Dingguo Changsheng Biotechnology Co. Ltd. (Beijing, China). The anti-mouse immunohistochemistry kit was purchased from Jingmei Inc. (Shanghai, China), anti-β-actin secondary antibody was purchased from Beijing Zhongshan Jinqiao Biotechnology Co. Ltd. (Beijing, China) and HRP-goat anti-mouse IgG was obtained from Beyotime Institute of Biotechnology (Shanghai, China).

#### Cell culture

Rhesus monkey retinal fovea vascular endothelial cells (RF/6A) were obtained from our laboratory (Department of Ophthalmology, The Second Xiangya Hospital, Xiangya School of Medicine, Central South University, Changsha). The RF/6A cells were maintained in DME containing 10% FBS. The cells were incubated with 5% CO_2_ at 37°C. The study was approved by the Institutional Review Board of The Second Xiangya Hospital, Central South University (Changsha, China).

#### Preparation of AGEs

Bovine serum albumin (BSA) was glycated by incubation with glucose-6-phosphate in phosphate-buffered saline (PBS) for 6 weeks at 37°C, as described previously (7). Dialyzed glycated protein was characterized based on fluorescence at 450 nm upon excitation at 390 nm using a fluorescence spectrometer (model LS-3B; Perkin-Elmer Corp., Norwalk, CT, USA). The endotoxin content in each sample was measured by the Limulus amebocyte lysate assay (E-Toxate; Sigma) and observed to be below detectable levels (0.2 ng/ml). For the control, non-glycated albumin consisted of the same initial preparations of albumin incubated at 37°C in the absence of sugar. The glycation process was performed twice, and the two AGE preparations yielded similar results.

#### Experimental groups

The cells were divided into two groups: the AGE experimental group and the non-glycated control group. The RF/6A cells were incubated with different concentrations of AGEs (0, 50, 100, 200, 400 and 800 mg/l) for 24 h.

#### Conditioned media VEGF measurements

The conditioned media VEGF levels were determined using a sandwich ELISA assay according to the manufacturer’s instructions. Briefly, cells were cultured for 24 h, the supernatant was removed and transferred to wells which were coated with a monoclonal antibody to VEGF. VEGF proteins levels were normalized to cell counts.

#### Detection of HIF-1α protein expression

Briefly, after the cells were cultured for 24 h, the culture solution was removed and cells were washed in PBS three times. The cells were fixed using 4% paraformaldehyde for 10 min. The cells were then incubated with polyclonal antibody to HIF-1α (1:100) and stained with DAB.

#### Western blot assay of HIF-1α protein

After the cells were cultured with various concentrations of AGEs for 24 h, the culture solution was discarded and cells were washed with PBS three times. The cells were scraped and diverted into a 1.5 ml microtube which contained RIPA buffer with protease inhibitor. After being put on ice for 3 min, the mixture was agitated, dissolved and put on ice for a further 30 min. The mixture was centrifuged at 4°C for 25 min [5,220 × g; Microcentrifuge 5415R (5415D); Eppendorf, Stevenage, UK], the top clear liquid layer was removed and western blotting was performed in triplicate.

#### Statistical analysis

Values were expressed as the mean ± standard deviation (SD). Statistical analyses were performed using SPSS software V11.5 (SPSS, Inc., Chicago, IL, USA). Continuous data were analyzed by the Levene method. The significance of a difference between groups was evaluated using one-way ANOVA with a post hoc Student-Newman-Keuls multiple comparisons test. The experimental and control groups were compared using a matched Student’s t-test. The correlation between VEGF and HIF-1α expression was analyzed with double variable regression and correlation analysis. P<0.05 was considered to indicate a statistically significant result.

## Results

### 

#### Morphological observation of RF/6A cells

**Figure 1:**
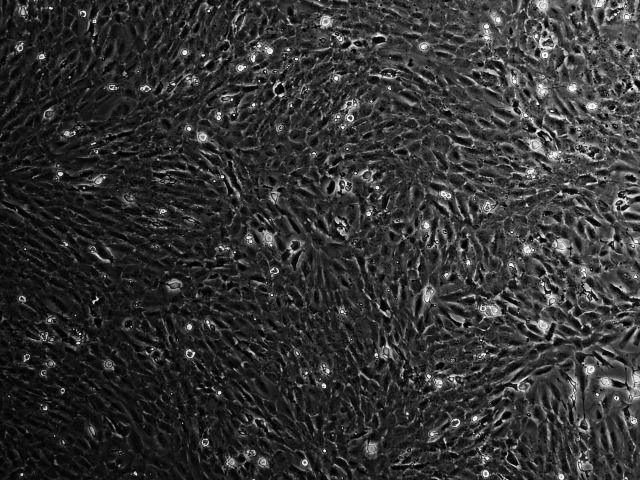
Morphology of confluent RF/6A cells (untreated, magnification ×40).

RF/6A cells are a type of monolayer-adherent cell. Under a microscope, the cells represented two different shapes. One type of cell was short and ‘shuttle-like’ with a diminished cell volume. In addition, the cytoplasm was particularly reflective, presented a typical ‘cobblestone’ appearance and was small in number. The other type of cell had a larger volume and was polygonal. This type of cell grew well, was greater in number, had extensive cytoplasm and one or more nucleoli (Fig. 1).

#### Changes in VEGF protein expression

**Figure 2:**
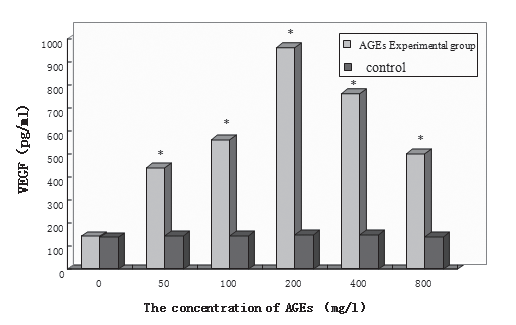
VEGF expression of RF/6A cells, ^*^P<0.05 vs. control group. VEGF, vascular endothelial growth factor; AGEs, advanced glycation end products.

As shown in Fig. 2, the non-glycated control groups secreted low levels of VEGF and there was no statistical significance between the cells treated with different concentrations of non-glycated albumin (P>0.05). By contrast, VEGF secretion was significantly higher in all AGE experimental groups compared with the control groups (P<0.05). In addition, VEGF secretion increased as the AGE concentration increased and the VEGF level reached its highest point at 200 mg/l AGE. At higher concentrations of AGEs, the VEGF secretion gradually decreased, but there remained a significant difference compared with the control group (P<0.05). In summary, VEGF expression was AGE concentration-dependent.

### HIF-1α protein expression in RF/6A cells

#### Immunocytochemical analysis of HIF-1α protein expression

**Figure 3:**
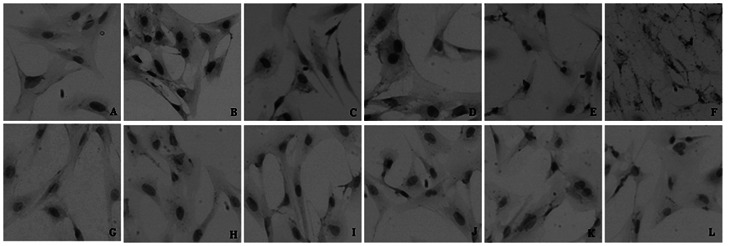
Expression of HIF-1α in RF/6A cells by immunocytochemical staining analysis. (A) Blank control group; (B–F) 50, 100, 200, 400 and 800 mg/l AGEs, respectively. (G) Negative control group; (H–L) contain 50, 100, 200, 400 and 800 mg/l non-glycated control group, respectively (magnification x200). AGEs, advanced glycation end products; HIF-1α, hypoxia-inducible factor-1α; blank control, AGEs were replaced by PBS; negative control, first antibody was replaced by PBS.

As shown in Fig. 3, HIF-1α protein expression was observed in the AGE experimental groups, where the majority of HIF-1α was located in the nucleus and a small quantity was located in the cytoplasm, which was significantly different from the control group (P<0.05). However, no significant differences (P>0.05) were observed among the experimental groups. In addition, the difference in HIF-1α expression between the AGE group and blank control group was not statistically significant (P>0.05). The non-glycated control groups had low HIF-1α protein expression levels, and there were no significant differences (P>0.05) among the non-glycated control groups. However, the HIF-1α expression levels of the AGE experimental groups were significantly higher compared with those of the non-glycated control groups.

#### Western blot analysis of HIF-1α protein expression

**Figure 4:**
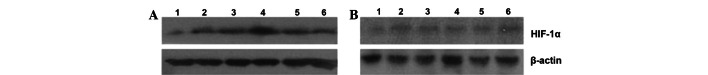
Western blot analysis of HIF-1α protein expression in RF/6A cells. (A) AGE experimental groups; (B) non-glycated control groups. Lanes 1, 2, 3, 4, 5 and 6 represent 0 (blank control group), 50, 100, 200, 400 and 800 mg/l AGEs or non-glycated albumin, respectively. AGEs, advanced glycation end products; HIF-1α, hypoxia-inducible factor-1α.

In the present study, we also detected the HIF-1α protein expression by western blot analysis. As shown in Fig. 4, the level of HIF-1α expression was lower in all non-glycated control groups than in the AGE experimental groups. However, different levels of HIF-1α protein expression were detected, and the HIF-1α expression level increased along with the AGE concentration. The maximum level of HIF-1α expression occurred when the cells were treated with 200 mg/l AGEs. The HIF-1α expression level then decreased with increasing AGE concentration. These results were consistent with VEGF expression in the AGE experimental groups.

**Figure 5:**
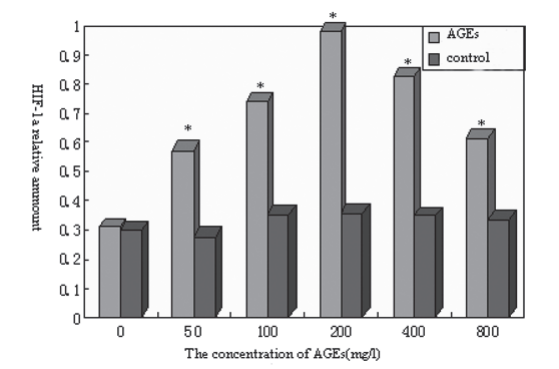
HIF-1α protein expression in RF/6A cells at different concentration of AGEs. ^*^P<0.05, vs. control group. AGEs, advanced glycation end products; HIF-1α, hypoxia-inducible factor-1α.

As shown in Fig. 5, the levels of HIF-1α protein expression in the non-glycated control groups were low, and no significant difference was observed among the control groups (P>0.05). However, the level of HIF-1α expression was significantly higher in all AGE experimental groups than in the control groups (P<0.05). Accordingly, as the AGE concentration increased, HIF-1α protein expression was promoted; the maximum HIF-1α expression occurred when the AGE concentration was 200 mg/l. The expression level then decreased, but with no significant difference (P>0.05). In summary, these results suggest that the HIF-1α expression level was dependent on AGE concentration.

#### Association between VEGF and HIF-1α expression of RF/6A cells with different concentrations of AGEs

The association between VEGF and HIF-1α was analyzed by a bivariate vector autoregressive model and correlation analysis. The results showed there was a positive correlation between VEGF and HIF-1α, and the regression equation was y = 1198.2× − 241.78, R=0.9881, P<0.01.

## Discussion

ssThe HIF-1 transcription factor mediates adaptive responses to alterations in tissue oxygenation. HIF-1 is a heterodimer that consists of two subunits; HIF-1β which is constitutively expressed and HIF-1α which is highly regulated. The level of HIF-1α expression is determined by the rates of protein synthesis and degradation. The synthesis of HIF-1α is regulated by O_2_-independent mechanisms. HIF-1α degradation is regulated mainly via O_2_-dependent mechanisms (11). A previous study indicated that HIF-1α expression is increased in DR mice compared with wild type mice, which shows that HIF-1α plays an important role in DR pathogenesis (12). Another study (13) has shown that HIF-1α mRNA and protein expression levels are increased in human breast cancer tissue. In the present study using AGEs, the majority of HIF-1α expression was located in the cell nucleus and less in the cytoplasm. The HIF-1α protein expression level was increased by AGE in a concentration-dependent manner, and the levels in the AGE groups were significantly different (P<0.05) from those in the control groups.

HIF-1 activates the transcription of genes that are involved in angiogenesis, which enables the body to rectify the oxygen deficit. VEGF is the most significant stimulating factor for vascularization and is the important target gene of HIF-1 (14). Previous studies have demonstrated that the HIF-1α and VEGF levels in the vitreous humor of DR patients and retinas of DR animals were increased, and that there is a correlation between the two (15,16). In the current study, HIF-1α and VEGF levels were observed to be increased markedly in RF/6A cells treated with AGEs, and the increase occurred in an AGE concentration-dependent manner. HIF-1α and VEGF expression levels increased gradually with increasing AGE concentration. The maximum level of protein expression appeared when the AGE concentration reached 200 mg/l. However, the expression of the two proteins decreased with further increase of the AGE concentration. A positive correlation was identified in our study. We suggest that the cell surface receptors reach a saturated state following combination with AGEs, which results in surplus AGEs that have no effect. In addition, excess AGEs are toxic to endothelial cells, so this result is associated with necrocytosis or apoptosis.

In conclusion, AGEs cause ischemia and oxygen deficit in retinal tissue, and this results in vascular leakage, vascular endothelial cell hyperplasia and neovascularization through increasing HIF-1α and following activation of the transcription of genes that are involved in angiogenesis such as VEGF. These results indicate that inhibition of the HIF-1 pathway may be a novel approach for the prevention of neovascularization in DR.
